# Chemical and Metabolic Controls on Dihydroxyacetone Metabolism Lead to Suboptimal Growth of Escherichia coli

**DOI:** 10.1128/AEM.00768-19

**Published:** 2019-07-18

**Authors:** Camille Peiro, Pierre Millard, Alessandro de Simone, Edern Cahoreau, Lindsay Peyriga, Brice Enjalbert, Stéphanie Heux

**Affiliations:** a LISBP, Université de Toulouse, CNRS, INRA, INSA, Toulouse, France; University of Tartu

**Keywords:** dihydroxyacetone, *Escherichia coli*, carbon metabolism, metabolic modeling

## Abstract

In this work, we shed light on the metabolism of dihydroxyacetone (DHA), a versatile, ubiquitous, and important intermediate for various chemicals in industry, by analyzing its metabolism at the system level in Escherichia coli. Using constraint-based modeling, we show that the growth of E. coli on DHA is suboptimal and identify the potential causes. Nuclear magnetic resonance analysis shows that DHA is degraded nonenzymatically into substrates known to be unfavorable to high growth rates. Transcriptomic analysis reveals that DHA promotes genes involved in biofilm formation, which may reduce the bacterial growth rate. Functional analysis of the genes involved in DHA metabolism proves that under the aerobic conditions used in this study, DHA is mainly assimilated via the dihydroxyacetone kinase pathway. In addition, these results show that the alternative routes of DHA assimilation (i.e., the glycerol and fructose-6-phosphate aldolase pathways) are not fully activated under our conditions because of anaerobically mediated hierarchical control. These pathways are therefore certainly unable to sustain fluxes as high as the ones predicted *in silico* for optimal aerobic growth on DHA. Overexpressing some of the genes in these pathways releases these constraints and restores the predicted optimal growth on DHA.

**IMPORTANCE** DHA is an attractive triose molecule with a wide range of applications, notably in cosmetics and the food and pharmaceutical industries. DHA is found in many species, from microorganisms to humans, and can be used by Escherichia coli as a growth substrate. However, knowledge about the mechanisms and regulation of this process is currently lacking, motivating our investigation of DHA metabolism in E. coli. We show that under aerobic conditions, E. coli growth on DHA is far from optimal and is hindered by chemical, hierarchical, and possibly allosteric constraints. We show that optimal growth on DHA can be restored by releasing the hierarchical constraint. These results improve our understanding of DHA metabolism and are likely to help unlock biotechnological applications involving DHA as an intermediate, such as the bioconversion of glycerol or C_1_ substrates into value-added chemicals.

## Abstract (precis)

DHA is an attractive triose molecule with a wide range of applications, notably in cosmetics and the food and pharmaceutical industries. DHA is found in many species, from microorganisms to humans, and can be used by Escherichia coli as a growth substrate. However, knowledge about the mechanisms and regulation of this process is currently lacking, motivating our investigation of DHA metabolism in E. coli. We show that under aerobic conditions, E. coli growth on DHA is far from optimal and is hindered by chemical, hierarchical, and possibly allosteric constraints. We show that optimal growth on DHA can be restored by releasing the hierarchical constraint. These results improve our understanding of DHA metabolism and are likely to help unlock biotechnological applications involving DHA as an intermediate, such as the bioconversion of glycerol or C_1_ substrates into value-added chemicals.

## INTRODUCTION

Dihydroxyacetone (DHA) is an attractive molecule that is used as a final product in a wide range of industries (i.e., the food, cosmetic, and pharmaceutical industries) (1–3) or as a growth substrate for various microorganisms (4–6). DHA is a ubiquitous molecule found in all domains. A physiological product of the body naturally present in human urine (7), it also plays a role in the osmoregulation of yeast and algae (8). DHA is produced as an intermediate of various metabolic pathways. In methylotrophic yeast, DHA is a key intermediate in methanol assimilation (9, 10), while in bacteria, DHA is produced by aldol cleavage of the glycolytic intermediary fructose 6-phosphate (11) or by oxidation of glycerol, the latter process being the basis for the current industrial production of DHA by Gluconobacter oxydans (12). DHA is also a highly reactive molecule, and its accumulation is presumed to be toxic. Indeed, DHA can react with DNA and proteins in Maillard-type reactions and thereby alter cell heredity (13, 14). In addition, DHA is unstable and can be converted nonenzymatically into several molecular species, among which is the highly toxic methylglyoxal (15–18).

Escherichia coli can metabolize DHA aerobically through at least three different metabolic pathways: (i) the dihydroxyacetone kinase (DAK) pathway, (ii) the glycerol (GLD) pathway, and (iii) the fructose-6-phosphate (FSA) pathway (17). The DAK pathway is named after dihydroxyacetone kinase, encoded by the *dhaKLM* operon. This operon is controlled by DhaR, a transcription factor activated by DHA (19). DhaKLM is composed of three subunits (DhaK, DhaL, and DhaM) and phosphorylates DHA to dihydroxyacetone phosphate (DHAP), an intermediate in the glycolytic pathway. DhaKLM has a high affinity for DHA (*K_m_* of <6 μM) and a catalytic constant (*k*_cat_) of 4.8 s^−1^ (20). This kinase resembles a phosphotransferase system (PTS) that uses phosphoenolpyruvate (PEP) as a phosphoryl donor (21). The DhaM subunit is first multiphosphorylated before the DhaL subunit uses the phosphate to convert ADP into ATP. Finally, this ATP is used directly by the DhaK subunit to phosphorylate DHA and form DHAP (22). The fact that *dhaK* transposon insertion prevents the growth of E. coli on DHA (23) indicates that DhaKLM is essential for this process (23).

The GLD pathway involves glycerol dehydrogenase (GldA) and glycerol kinase (GlpK). Hydroxyacetone induces *gldA* expression in the stationary phase (24) while the transcription of *glpK* is regulated by catabolite repression and by glycerol and glycerol-3-phosphate via the transcriptional repressor GlpR (25). During anaerobic glycerol fermentation, GldA enables the formation of DHA from glycerol (4), whereas under aerobic conditions, GldA enables the reverse process, i.e., the formation of glycerol from DHA (17, 26). GldA has a greater affinity for DHA (*K_m_* of 0.3 mM) than for glycerol (*K_m_* of 56 mM) (17), but the catalytic rate constants are close (*k*_cat_ of 17.2 and 22.4 s^−1^, respectively). It has been proposed that the primary *in vivo* role of GldA is the removal of surplus dihydroxyacetone to limit its toxic effects (17). However, DhaKLM and GldA have been shown to constitute a fermentative route for the conversion of glycerol to glycolytic intermediates (27), demonstrating that GldA is also involved in fermentation of glycerol (4). Once formed from DHA, glycerol is phosphorylated by GlpK to form glycerol-3-phosphate, which is central for lipid biosynthesis. GlpK has been shown to play a crucial role in DHA assimilation in various microorganisms (28–31). In Haloferax volcanii, for instance, the deletion of *glpK* has a higher impact on growth than does *dhaKLM* deletion, and mutants with both genes deleted do not grow on DHA (29). It has been hypothesized that in these organisms, GlpK can either phosphorylate DHA into DHAP or control the transport of DHA into the cells (28, 29). In E. coli, *in vitro* results indicate that GlpK is able to use DHA as a substrate with a *K_m_* value of 0.5 mM (32); however, its *in vivo* implication in DHA metabolism has never been studied.

The central enzyme in the FSA pathway is fructose-6-aldolase, which condenses DHA with glyceraldehyde-3-phosphate (GAP) to form fructose-6-phosphate (F6P). Two different genes in E. coli (*fsaA* and *fsaB* or *mipB* and *talC*) code for FsaA and FsaB, respectively. These two enzymes share 70% identity and have similar affinities for DHA (*K_m_* of 32 mM for FsaA and 27 mM for FsaB), but FsaA has a higher catalytic constant (*k*_cat_) for DHA (116 s^−1^ versus 41 s^−1^) (11). The physiological role of FsaA in E. coli (33) and its regulation remain unknown. However, *fsaB* is in the same operon as *gldA* and *ptsA*, suggesting that *fsaB* is metabolically associated with DHA (17). The role of this operon and its regulation are not completely clear. However, PtsA has been identified as a component of a putative PTS system involved in anaerobic fructose catabolism (34). In addition, higher expression of *gldA*, *fsaA*, and *fsaB* has been observed in wild-type E. coli grown on glucose during a transition from aerobic to anaerobic cultivation (35).

While several pathways potentially can support DHA catabolism in E. coli, which ones are actually involved during growth on DHA remains unclear, and their regulation is poorly characterized. This missing information would help unravel the physiological significance of this versatile, ubiquitous, and high-potential metabolite. The objective of our study was to investigate DHA metabolism in E. coli using system-level analysis. We first predicted the metabolic fate of DHA using a genome-scale model of E. coli to evaluate its metabolic capabilities. We then used transcriptomic analysis to identify “genes that matter” for growth on DHA. Based on these results, we characterized the physiology effect of overexpression and deletion of genes involved in DHA metabolism, and we propose mechanisms that would explain the fate of DHA in E. coli.

## RESULTS AND DISCUSSION

### DHA metabolism in E. coli involves a complex network of chemical and biological processes.

**FIG 1:**
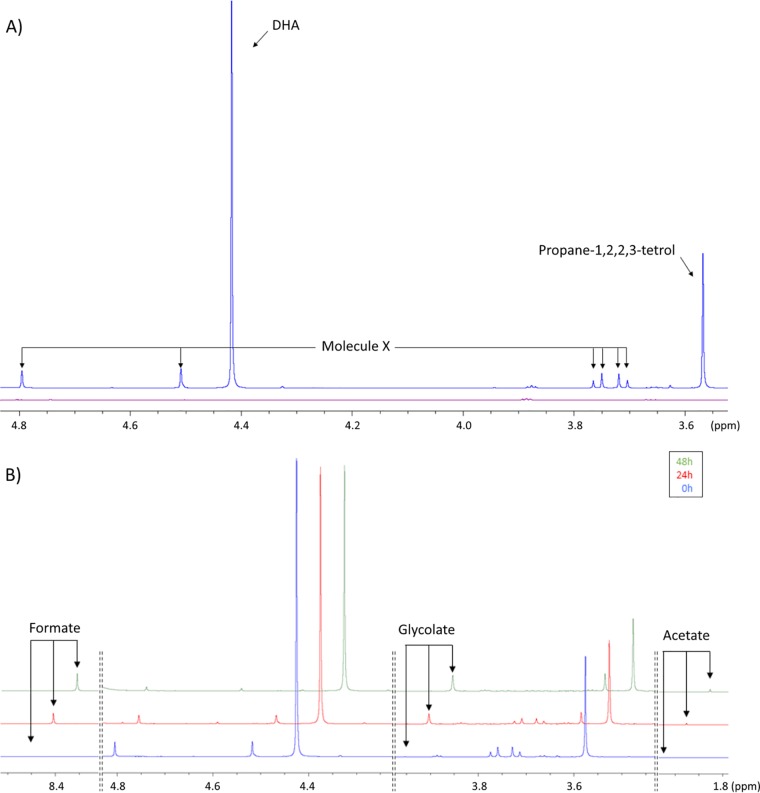
Nonenzymatic transformation of DHA in M9 medium with 5× diluted salts. (A) NMR spectrum overlay of nonincubated modified M9 medium with (blue) or without (purple) 15 mM DHA. (B) Kinetics of nonenzymatic transformation of DHA in modified M9 medium after 0 h (blue), 24 h (red), and 48 h (green) of incubation at 37°C. The three spectra are shown with a vertical step of 10% and a horizontal offstep of 0.05 ppm.

We carried out cultivation experiments to study the global behavior of E. coli during growth on DHA, monitoring the concentrations of biomass and extracellular metabolites as a function of time. DHA is an unstable compound that can be interconverted into different forms when dissolved in water (18) or autoxidized by Fenton’s reaction to form glycolate, other short-chained carbohydrates, and organic acids upon incubation (15, 16). We therefore determined beforehand whether nonenzymatic conversions of DHA could occur under our conditions. Noninoculated M9 minimal medium containing 15 mM DHA was incubated at 37°C for 48 h, and samples were collected after 0, 24, and 48 h of incubation and analyzed by ^1^H-nuclear magnetic resonance (NMR) (Fig. 1). Since DHA is a symmetrical molecule and all the detectable protons from the two CH_2_ moieties are equivalent, the ^1^H-NMR spectrum of pure DHA is expected to contain a singlet at 4.4 ppm. However, several signals were detected in the medium at time zero (Fig. 1A). None of these peaks were observed in a sample containing M9 medium without DHA (Fig. 1A), suggesting that they originated from the DHA solution. Two-dimensional (2D) NMR analysis (see Fig. S1 in the supplemental material) shows that the signal at 3.6 ppm corresponds to a hydrated form of DHA, propane-1,2,2,3-tetrol (or dihydroxyacetone monomer hydrate), as previously reported (18). Under our conditions, the proportions of nonhydrated and hydrated DHA were 65% ± 2% and 21% ± 2%, respectively. The remaining 14% ± 2% corresponds to the signals at 4.8, 4.7 (both singlets), and 3.7 (AB system) ppm, all originating from the same unidentified molecule X, which appeared after freezing/thawing the samples. Unfortunately, we were unable to identify the compound corresponding to these peaks.

Upon incubation of the noninoculated medium, the peaks from DHA, propane-1,2,2,3-tetrol, and molecule X decreased in intensity, while additional signals appeared that were not seen with the initial spectrum (Fig. 1B). This phenomenon was influenced by the amount of salts (i.e., Na_2_HPO_4_, KH_2_PO_4_, NaCl, and NH_4_Cl) in the medium. At the salt concentration typically used for the M9 medium (36), 82% of DHA was degraded after 48 h, while only 34% was degraded when Na_2_HPO_4_, KH_2_PO_4_, NaCl, and NH_4_Cl were diluted five times (data not shown). Thus, a 5× reduced salt concentration was used for all the subsequent experiments. In this modified M9 medium, DHA was mainly converted into formate, glycolate, and acetate (Fig. 1B and Fig. S2). After 39 h of incubation, 27% of the DHA was converted into formate (36%), glycolate (26%), and acetate (1.3%). The nonlinear DHA concentration profile was fitted assuming first-order kinetics. The excellent agreement between experimental and fitted curves (Pearson’s *r* of >0.99) validates the proposed degradation kinetics; the estimated degradation rate constant is 0.0086 ± 0.0010 h^−1^, corresponding to a DHA half-life of 116 h (Fig. S2).

In the inoculated medium, the peaks from both forms of DHA decreased monotonously over time (data not shown). The proportions of both forms remained constant during the experiment, suggesting that chemical equilibrium is faster than DHA uptake. No accumulation of glycolate, formate, or acetate was observed, indicating that these products of nonenzymatic DHA degradation are coconsumed with DHA by wild-type E. coli. In order to infer quantitative flux information from these data, we developed an R program, PhysioFit, a mathematical model to estimate the growth rate and exchange (uptake or production) fluxes. This model, which takes into account nonenzymatic degradation of substrates, is described in detail in Materials and Methods. This program calculated a growth rate of 0.15 ± 0.03 h^−1^ with a DHA uptake rate of 5.2 ± 0.19 mmol g_DW_^−1^ h^−1^ (corresponding to 15.6 Cmmol g_DW_^−1^ h^−1^; g_DW_ is grams of dry weight). Based on the nonenzymatic conversion yields of DHA, we estimated that DHA accounted for 74.3% of E. coli carbon uptake (expressed in Cmmol), with the remaining carbon taken up as formate (15.3%), glycolate (9.7%), and acetate (0.7%). This represents a specific uptake rate of 3.2, 1.0, and 0.1 mmol g_DW_^−1^ h^−1^, respectively (i.e., 3.2, 2.0, and 0.2 Cmmol g_DW_^−1^ h^−1^).

**FIG 2:**
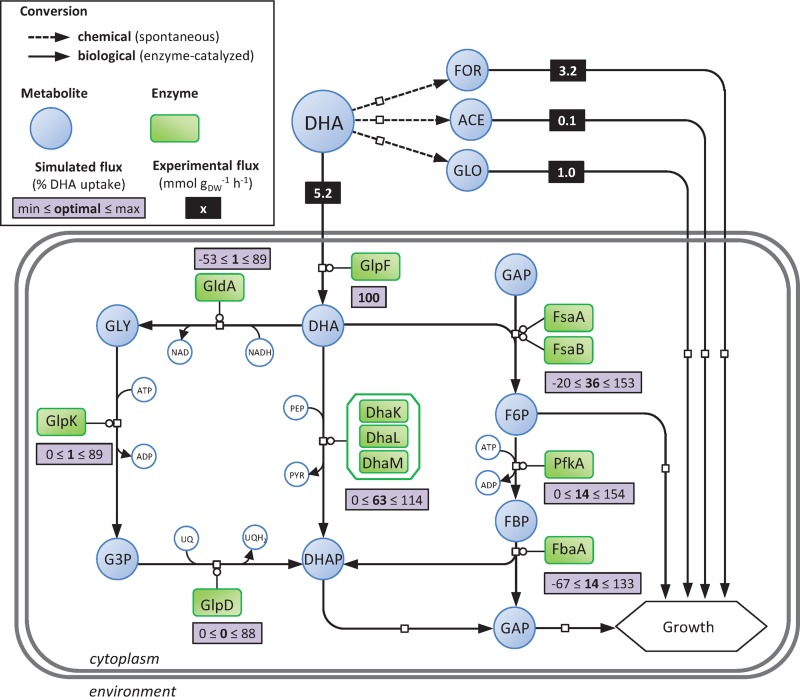
Experimental and simulated fluxes through DHA metabolism of E. coli. Gray rectangles give the optimal and the ranges of simulated fluxes. Optimal simulated fluxes were obtained using flux balance analysis constrained with experimental uptake fluxes of the wild-type strain grown on modified M9 media containing 15 mM DHA (i.e., values given in black rectangle). Ranges of simulated fluxes were obtained using flux variability analysis when growth rate is constrained to 95% of the optimal value and with experimental uptake fluxes. Experimental flux values are given in mmol g_DW_^−1^ h^−1^, and simulated flux values are given in percent relative to DHA uptake rate. Shown are dihydroxyacetone kinase enzymes (DhaK, DhaL, and DhaM), fructose-6-phosphate aldolase enzymes (FsaA and FsaB), glycerol dehydrogenase enzyme (GldA), glycerol facilitator (GlpF), glycerol-3-phosphate dehydrogenase enzyme (GlpD), glycerol kinase enzyme (GlpK), fructose-bisphosphate aldolase enzyme (FbaA), 6-phosphofructokinase enzyme (PfkA), dihydroxyacetone (DHA), formate (FOR), acetate (ACE), glycolate (GLO), dihydroxyacetone phosphate (DHAP), fructose-6-phosphate (F6P), fructose-1,6-bisphosphate (FBP), glyceraldehyde-3-phosphate (GAP), glycerol (GLY), glycerol-3-phosphate (G3P), phosphoenolpyruvate (PEP), pyruvate (PYR), ubiquinone (UQ), ubiquinol (UQH_2_).

Overall, these results show that an additional set of reactions (i.e., nonenzymatic conversion of DHA) occur under these condition (Fig. 2) that have to be taken into account when studying the metabolism of DHA.

### E. coli growth on DHA is robust but suboptimal.

Constraint-based metabolic models provide an attractive starting point for studying poorly characterized metabolisms, because they can predict growth rates and metabolite flows through a metabolic network with minimal *a priori* knowledge. We performed flux balance analysis (FBA) using a genome-scale E. coli model (iJO1366 [37]) to simulate the fate of DHA (Fig. 2). The only constraints used were the DHA, formate, glycolate, and acetate uptake rates of wild-type E. coli, calculated as detailed above. The model predicted a growth rate of 0.28 h^−1^, i.e., almost 2 times higher than the experimentally observed growth rate (0.15 h^−1^). Since FBA predictions assume optimal growth under stoichiometric and maximum uptake constraints, these results suggest that for some reason the system was not operating optimally *in vivo*. For optimal growth, the predicted model has 63% of the DHA converted into DHAP via the DAK-based pathway and 36% via the FSA pathway. The GLD-based pathway is only used to feed glycerol-3-phosphate for biomass synthesis (Fig. 2). Flux balance analysis computes an optimal objective value and a flux state that are consistent with that objective (and all the imposed constraints). While the objective value (i.e., the growth rate under our conditions) is unique, this can typically be supported by multiple flux states in genome-scale models. For this reason, we performed flux variability analysis (FVA) to find the minimum and maximum flux for each reaction in the network while maintaining growth at 95% of its optimal value (38). The results indicate that the fluxes through the GLD-, DAK-, and FSA-based pathways offer a wide range of possible values, including opposite fluxes (i.e., reverse reactions). These data suggest that, based solely on stoichiometric constraints, optimal growth does not in theory depend on any particular DHA metabolic pathway.

Overall, these data demonstrate flexible use of all the DHA catabolic pathways and suggest that this metabolism in E. coli is highly robust.

### DHA-induced genes involved in a biofilm growth state.

In order to gain insight into the regulation of DHA metabolism, we compared the global transcriptional responses of wild-type E. coli grown on DHA (Fig. S3) and glucose medium. This revealed that several genes related to mobility, adherence, and biofilm formation and stress were expressed differently between DHA and glucose growth (Table 1 and Data File S1). For instance, the *csg* genes, encoding curli fibers, which promote cell adhesion during biofilm formation (39), were expressed at a higher level (by a factor of 10 on average) during DHA growth. Conversely, genes involved in motility and flagellum assembly were less expressed (e.g., by factors of 25- and 12-fold on average for the *flg* and *fli* genes, respectively) (Table 1 and Data File S1), indicating that motility is repressed on DHA. Genes related to lipopolysaccharide (LPS) synthesis were expressed by a factor of 4 on DHA (Table 1 and Data File S1). LPSs are a major component of the outer leaflet of the outer membrane of most Gram-negative bacteria, contributing to their structural integrity. LPSs govern many of the biological interactions between cells and their environment (40) and play a crucial role in the anchoring process (41). Taken together, these data suggest that E. coli switches from planktonic growth on glucose to biofilm growth on DHA. This might contribute to the low growth rate of E. coli on DHA, since cells in biofilms are known to grow more slowly than in the planktonic mode (42).

**TABLE 1:** Functional classification of genes with statistically significant decreases and increases in mRNA level in E. coli strain BW25113^*a*^

Category and expression status	GO concerned	*P* value	Gene examples
Overexpressed			
Cell wall and adhesion	GO:0009103; lipopolysaccharide biosynthetic process	2.66E−14	*waaO*, *wbbJ*, *arnC*, *wcaB*, *waaB*, *waaJ*, *waaY*, *waaU*, *rfbA*, *rfbC*
	GO:0022610; biological adhesion	8.85E−12	*dgcZ*, *elfA*, *yadK*, *yehA*, *pgaC*, *yeeJ*, *csgB*, *csgF*, *csgE*, *csgG*
	GO:0043711; pilus organization	4.79E−10	*htrE*, *yehB*, *ybgQ*, *elfC*, *sfmD*, *ydeT*, *yqiG*, *fimD*, *sfmC*, *elfD*
Response to host	GO:0006952; defense response	4.50E−07	*casE*, *casD*, *casC*, *casB*, *casA*, *abpA*, *abpB*, *rzpD*, *rrrQ*, *rzpR*
	GO:0009243; O antigen biosynthetic process	4.86E−05	*rfbC*, *rfbD*, *rfbB*, *rfbA*, *wbbI*, *rfbX*
Response to acidic pH	GO:0010447; response to acidic pH	6.07E−05	*cadB*, *yjaA*, *hdeA*, *iraM*, *oxc*, *frc*, *glsA*, *yagU*, *evgS*, *hdeB*
Copper ion homeostasis	GO:0006878; cellular copper ion homeostasis	7.59E−04	*cusA*, *cusB*, *cusC*, *cusF*
Involved in glucarate catabolic process	GO:0019394; glucarate catabolic process	1.67E−04	*garKLR*, *gudD*
Underexpressed			
Locomotion	GO:0040011; locomotion	1.28E−24	*fliL*, *flgB*, *flgC*, *flgG*, *flgD*, *flgE*, *flgF*, *motA*, *fdrA*, *fliG*
Cellular respiration	GO:0045333; cellular respiration	6.09E−15	*frdB*, *hyaA*, *hyaB*, *pflD*, *fdoG*, *fdoH*, *frdA*, *frdC*, *frdD*, *hybB*
Primary metabolism	GO:0019321; pentose metabolic process	2.11E−10	*rbsD*, *rbsK*, *xylA*, *araD*, *araA*, *araB*, *xylF*, *xylG*, *xylH*, *rhaD*
	GO:0016052; carbohydrate catabolic process	3.19E−9	*treC*, *lacZ*, *melA*, *malS*, *gldA*, *hybA*, *gatY*, *uidA*, *gntK*, *gntP*, *gntT*
	GO:0008643; carbohydrate transport	4.84E−09	*kdgT*, *gntP*, *gntT*, *ptsG*, *yiaO*, *melB*, *rbsA*, *srlB*, *frwC*, *treB*
	GO:0071941; nitrogen cycle metabolic process	2.45E−08	*argG*, *argI*, *argF*, *carA*, *ygeW*, *napC*, *nirB*, *narG*, *nirD*, *nirC*
	GO:0006099; tricarboxylic acid cycle	5.99E−07	*sdhD*, *sdhC*, *sdhB*, *acnA*, *sucA*, *fumB*, *mdh*, *sdhA*, *sucD*, *sucC*

a mRNA levels were examined in E. coli strain BW25113 in modified M9-DHA medium and compared to values for M9 glucose medium (60). The Clusters of Orthologous Groups (COG) were used for grouping.

### The DAK pathway is central but not essential for DHA metabolism.

**FIG 3:**
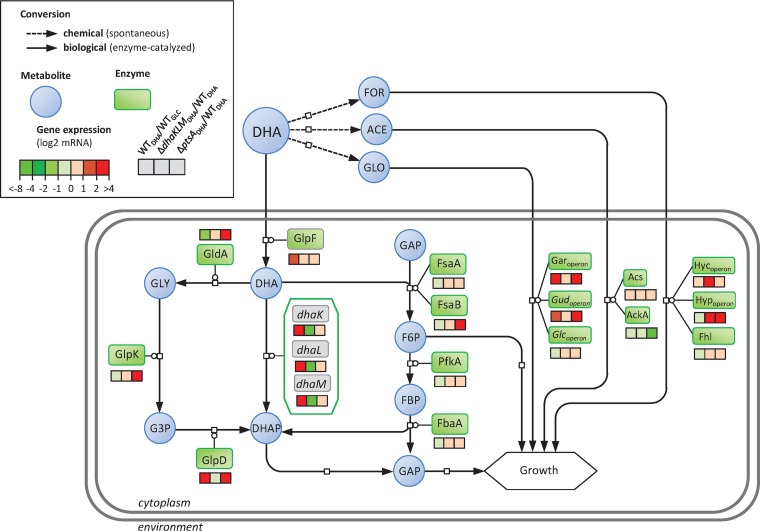
Change in gene expression in DHA metabolism of the wild-type strain, Δ*dhaKLM* strain, and Δ*ptsA* strain cultured on modified M9-DHA medium. For the wild-type strain (WT_DHA_), the fold changes in gene expression were calculated in reference to expression in an E. coli strain cultured in chemostat in M9-glucose medium at 0.1 h^−1^ (WT_GLC_). For the Δ*dhaKLM* (Δ*dhaKLM*_DHA_) and the Δ*ptsA* (Δ*ptsA*_DHA_) strains, the fold changes in gene expression were calculated in reference to expression in an E. coli strain cultured in modified M9-DHA medium. The *ptsA* gene in the Δ*ptsA* mutant was replaced by a kanamycin resistance cassette, leading to the overexpression of *gldA* and *fsaB*, which are part of the same operon. For all experiments, *n* = 2 biological replicates, and log_2_ values are given. Shown are dihydroxyacetone kinase genes (*dhaK*, *dhaL*, and *dhaM*), fructose-6-phosphate aldolase genes (*fsaA* and *fsaB*), glycerol dehydrogenase gene (*gldA*), glycerol facilitator (*glpF*), glycerol-3-phosphate dehydrogenase gene (*glpD*), glycerol kinase gene (*glpK*), fructose-bisphosphate aldolase gene (*fbaA)*, 6-phosphofructokinase gene (*pfkA*), glucarate operon (*gar*_operon_ and *gud*_operon_), glycolate operon (*glc*_operon_), acetyl-coenzyme A synthetase gene (*acs*), acetate kinase (*ackA*), formate hydrogenlyase system genes (*hy*c_operon_, *hyp*_operon_, and *fhl*), enzyme I gene (*ptsI*), dihydroxyacetone (DHA), formate (FOR), acetate (ACE), glycolate (GLO), dihydroxyacetone phosphate (DHAP), fructose-6-phosphate (F6P), fructose-1,6-bisphosphate (FBP), glyceraldehyde-3-phosphate (GAP), glycerol (GLY), and glycerol-3-phosphate (G3P).

**FIG 4:**
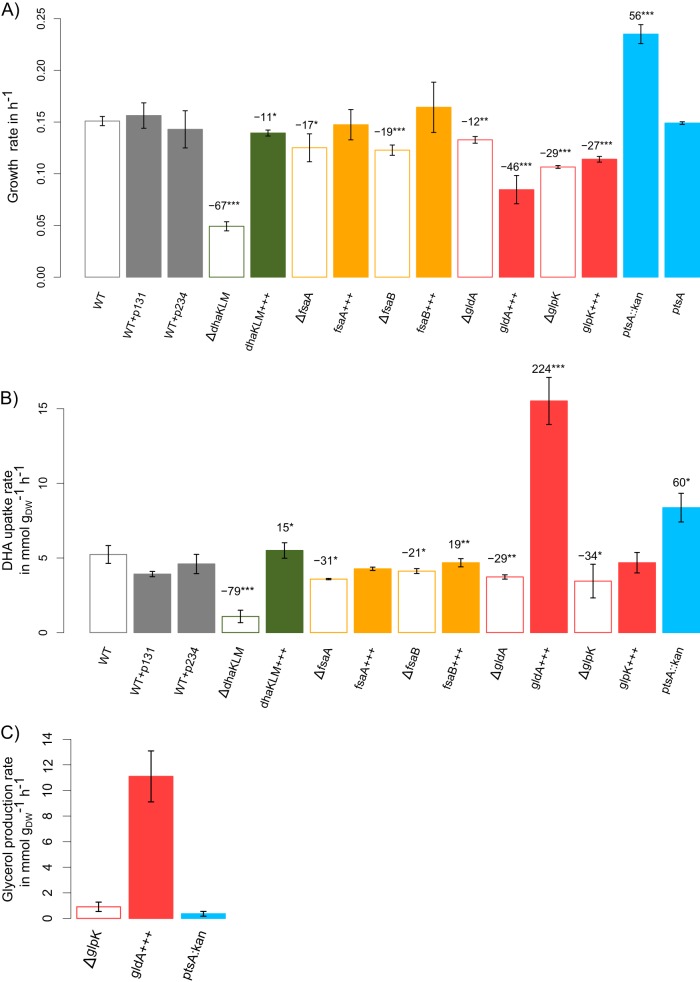
Growth rate (A), specific DHA uptake rate (B), and specific glycerol production rate (C) of the strains with deletions (Δ) and overexpression (+++) of genes involved in DHA metabolism (Table 2). Cells were grown on modified M9 medium with 15 mM DHA at 37°C and shaking at 220 rpm. Growth rate is given in h^−1^. DHA uptake rate and glycerol production rate are given in mmol g_DW_^−1^ h^−1^. PhysioFit was used to estimate growth and exchange rates. Data shown represent means and standard deviations (*n* = 3). *P* values were obtained using a *t* test comparing mutant strains with the WT control (***, *P* < 0.001; **, *P* < 0.01; *, *P* < 0.05). Refer to Table 2 for details on each strain.

The regulation of *dhaKLM* expression (19) and the properties of the enzyme (20) have led to the suggestion that the DAK pathway is important and even essential for growth on DHA in E. coli (23). To test this hypothesis, we investigated the expression levels of the genes involved in DHA metabolism in wild-type and Δ*dhaKLM* strains grown on DHA (Fig. 3 and Fig. S3) and studied the functional characteristics of DhaKLM (Fig. 4). Comparing the expression levels of wild-type strains grown on DHA versus glucose shows that the genes involved in glucarate metabolism (i.e., the *gar* and *gud* operons) are upregulated by a factor of about 5 on average (Fig. 3 and Data File S1), while those involved in glycolate metabolism (i.e., the *glc* operon) were not. As shown previously, DHA is spontaneously converted into glycolate, which is supposed to induce the *glc* genes (43); however, glycolate can also be recognized by the same transporter as glucarate (44) and catabolized using part of its metabolic pathway. The gene encoding the glycerol facilitator GlpF, a nonspecific channel protein capable of transporting straight-chain carbon compounds such as DHA, was upregulated about 3-fold, consistent with its putative role in DHA transport (23). As expected, both the *dhaKLM* operon and its transcription factor, *dhaR*, were strongly upregulated (Fig. 3 and Data File S1). However, the expression levels of *glpK*, *fsaA*, and *fsaB* were not affected, and *gldA* was downregulated by a factor of 2.5 (Fig. 3). Transcriptome analysis of the Δ*dhaKLM* and wild-type strains (Fig. 3) revealed that none of the genes of the FSA and GLD pathways were upregulated in the Δ*dhaKLM* strain, demonstrating that there is no compensatory activation of these pathways. Interestingly, the *hyc* and *hyp* operons and Fhl enzymes, involved in the production of dihydrogen and carbon dioxide in formate metabolism (45) and activated by formate, were upregulated by a factor of 10 (Fig. 3 and Data File S1). This points to the presence of formate in the medium, which is consistent with the nonenzymatic degradation of DHA into formate, and suggests that Fhl enzymes allow the Δ*dhaKLM* strain to convert formate from the medium and use the hydrogen as an electron donor for respiration (46). Deletion of *dhaKLM* resulted in a significant and strong reduction of the growth rate and a shift in the substrate profiles compared with those of the control strain (Fig. S3 and S4A), while complementation of the deleted genes restored normal growth (Fig. S4). Furthermore, in this strain the specific DHA uptake rate was strongly reduced. When *dhaKLM* was overexpressed (i.e., *dhaKLM*+++), the specific DHA uptake rate increased slightly, by 15% compared with that of the wild type, but the growth rate did not (Fig. 4). Taken together, these data support the hypothesis that DhaKLM plays a crucial role in DHA utilization, in agreement with the transcriptomics data. However, under our conditions, and contrary to previous observations in E. coli (23), inactivation of *dhaKLM* was not lethal. This is because the metabolism of DHA is shifted to formate, glycolate, and acetate metabolism (due to the instability of DHA) in this strain. Based on the nonenzymatic conversion yields of DHA into formate, glycolate, and acetate determined above, we estimate that formate accounted for 38.5% of the Δ*dhaKLM* strain’s carbon uptake (expressed in Cmmol), while DHA, glycolate, and acetate accounted for 34, 25.7, and 1.6%, respectively.

Overall these data show that the operon *dhaKLM* is predominantly expressed during growth on DHA, while the genes encoding the GLD and FSA pathways are not transcriptionally activated, even to rescue the functionality of a strain with *dhaKLM* deleted. Instead, this strain survives on DHA’s degradation products, leaving the alternative catabolic pathways dormant.

### The GLD and FSA pathways are functionally involved in DHA metabolism.

To assess whether the GLD and FSA pathways are functionally involved in DHA metabolism even if their transcription is not activated on DHA, we performed functional analysis of the genes involved in these pathways. The results obtained for all the deletion or overexpressing strains (Table 2) grown on minimal medium containing DHA are shown in Fig. 4. Inactivation of *gldA*, *glpK*, *fsaA*, and *fsaB* resulted in significantly slower growth than that of the control strain (Fig. 4A), and complementation of the deleted genes restored the growth rate (Fig. S4). After Δ*dhaKLM*, the gene deletion that had the greatest impact on the growth rate and the specific DHA uptake rate was Δ*glpK*, reducing them by 29 and 34%, respectively (Fig. 4A and B). In H. volcanii (29), GlpK has a central role in DHA metabolism because it can directly phosphorylate DHA to DHAP using ATP. If GlpK could act as an ATP-dependent DHA kinase in E. coli, its overexpression would improve the fitness of the cells. Indeed, the conversion of DHAP to pyruvate generates two ATPs and only one PEP, and a doubling of the level of phosphate donors (ATP molecules) in the cytoplasm should support higher ATP-dependent DHA kinase activity than PEP-dependent DHA kinase and, thus, better growth. This has indeed been observed recently in an E. coli strain that overexpresses an ATP-dependent DHA kinase during anaerobic growth on DHA (47). However, this was not observed under our aerobic conditions, i.e., overexpressing *glpK* (i.e., *glpK*+++) decreased the growth rate (Fig. 4A), suggesting that GlpK does not act *in vivo* as an ATP-dependent DHA kinase enzyme. In addition, the production of glycerol observed in the Δ*glpK* strain (Fig. 4C) clearly indicates that GlpK is involved in glycerol phosphorylation rather than in DHA phosphorylation. Results obtained for the strain overexpressing *gldA* (i.e., *gldA*+++) also support this hypothesis. In this strain, glycerol was produced at almost the same rate that DHA was taken up (Fig. 4C) and started to be consumed only when DHA was exhausted (Fig. S5). This suggests that DHA has an effect *in vivo* on glycerol utilization. In E. coli, the catalytic activity of GlpK is inhibited allosterically by enzyme IIA from the glucose PTS system (48). It could also be inhibited allosterically by enzyme IIA from the DHA PTS system (i.e., DhaM) (22). Deletion of *gldA* (i.e, Δ*gldA*) reduced both the growth and the specific DHA uptake rate (Fig. 4A and B), most probably because the production of glycerol-3-phosphate, which is essential for biomass synthesis, is provided by just one pathway (i.e., from DHAP via glycerol-3-phosphate dehydrogenase, encoded by *gpsA*). Overexpressing *gldA* more than doubled the specific DHA uptake rate (Fig. 4B), which is consistent with its high affinity for DHA. However, the growth rate was not improved (Fig. 4A) because of a bottleneck at the GlpK level, possibly due to allosteric regulation by DHA.

**TABLE 2:** Bacterial strains, plasmids, and primers used in this study

Plasmid, bacterial strain, or primer	Gene accession no.	Description (genotype and/or relevant characteristic[s]) or sequence of oligonucleotide primer	Reference or source
E. coli bacterial strains			
WT		BW25113; *rrnB3* Δ*lacZ4787 hsdR514* Δ(*araBAD*)*567* Δ(*rhaBAD*)*568 rph-1*	49
Δ*fsaA*	b0825	BW25113 Δ*fsaA*::*frt*	This study
Δ*fsaB*	b3946	BW25113 Δ*fsaB*::*frt*	This study
Δ*gldA*	b3945	BW25113 Δ*gldA*::*frt*	This study
Δ*glpK*	b3926	BW25113 Δ*glpK*::*frt*	This study
Δ*dhaKLM*	b1200, b1199, b1198	BW25113 Δ*dhaKLM*::*frt*	This study
Δ*ptsA*::*kan*	b3947	BW25113 Δ*ptsA*::*kan*	49
Δ*ptsA*	b3947	BW25113 Δ*ptsA*::*frt*	This study
WT+p131		BW25113 + pSEVA131	This study
WT+p234		BW25113 + pSEVA234	This study
*fsaA*+++	b0825	BW25113 + pSEVA234-fsaA	This study
*fsaB*+++	b3946	BW25113 + pSEVA131-fsaB	This study
*gldA*+++	b3945	BW25113 + pSEVA131-gldA	This study
*glpK*+++	b3926	BW25113 + pSEVA131-glpK	This study
*dhaKLM*+++	b1200, b1199, b1198	BW25113 + pSEVA131-dhaKLM	This study
Δ*fsaA*+++	b0825	∆*fsaA* + pSEVA234-fsaA	This study
Δ*fsaB*+++	b3946	∆*fsaB* + pSEVA131-fsaB	This study
Δ*gldA*+++	b3945	∆*gldA* + pSEVA131-gldA	This study
Δ*glpK*+++	b3926	∆*glpK* + pSEVA131-glpK	This study
Δ*dhaKLM*+++	b1200, b1199, b1198	∆*dhaKLM* + pSEVA131-dhaKLM	This study
Plasmids			
pSEVA131		Medium copy number, *lacI*^q^/Ptrc promotor, pBBR1 ori, Amp^r^; original pSEVA131 plasmid does not contain a promotor	66
pSEVA234		Medium copy number, *lacI*^q^/Ptrc promotor, pBBR1 ori, Km^r^	67
pSEVA131-dhaKLM		Derivative of pSEVA-131 containing *dhaKLM* operon; used to overexpress *dhaKLM* in BW25113	This study
pSEVA131-fsaB		Derivative of pSEVA-131 containing *fsaB* gene; used to overexpress *fsaB* in BW25113	This study
pSEVA131-gldA		Derivative of pSEVA-131 containing *gldA* gene; used to overexpress *gldA* in BW25113	This study
pSEVA131-glpK		Derivative of pSEVA-131 containing *glpK* gene; used to overexpress *glpK* in BW25113	This study
pSEVA234-fsaA		Derivative of pSEVA-234 containing *fsaA* gene; used to overexpress *fsaA* in BW25113	This study
Primers			
dhaKLM_knockout_F		CGTGTCGTTGAACATCATCCATGCCCTACCGTAATTGCTGGAGCAAAATAGTGTAGGCTGGAGCTGCTTC	
dhaKLM_knockout_R		CATCAGAACGATGCCATCCGAACAGTGGCTTAACCCTGACGGTTGAAACGCATATGAATATCCTCCTTAG	
glpK_knockout_F		TCCTTCAGAACAAAAAGCTTCGCTGTAATATGACTACGGGACAATTAA ACGTGTAGGCTGGAGCTGCTTC	
glpK_knockout_R		ACGTTTCGGGACTACCGGATGCGGCATAAACGCTTCATTCGGCATTTACACATATGAATATCCTCCTTAG	
Cm_F		AATCGTCGTGGTATTCACTCC	

Finally, the deletions of *fsaA* and *fsaB* (i.e., Δ*fsaA* and Δ*fsaB*) reduced both the growth rate and the specific DHA uptake rate (Fig. 4A and B). While their overexpression (i.e., *fsaA*+++ and *fsaB*+++) did not have a significant effect on the growth rate compared to that of the wild type, *fsaB* overexpression was associated with a significant increase in the DHA uptake rate. This demonstrates that FsaA and FsaB are involved in DHA utilization and suggests that the concentration of FsaB is limited in the wild-type strain.

Overall, these results show that GLD and FSA are both functionally involved in DHA metabolism but that their action is restricted. A number of strands of evidence suggest that *fsaB* and *gldA* are both anaerobic genes whose transcription is activated under anaerobic conditions (27, 34, 35). This would explain why these genes were not highly expressed under our aerobic conditions and, thus, why these pathways were only weakly used for DHA assimilation.

### Overexpressing the GLD and FSA pathways leads to optimal growth on DHA.

We used a *ptsA* mutant from the KEIO collection (49) to test whether optimal DHA growth could be restored by overexpressing the FSA- and GLD-based pathways. The *ptsA* gene in this mutant is replaced by a kanamycin resistance cassette, leaving *gldA* and *fsaB* expression under the control of the kanamycin promoter. As a result, *gldA* and *fsaB* were overexpressed 28- and 123-fold, respectively, compared with expression of the wild-type strain (Fig. 2 and Fig. S3 and Data File S1). Interestingly, in the Δ*ptsA*::*kan* mutant, *glpK* was also upregulated (by a factor of 10), making the GLD pathway fully activated. Furthermore, the Δ*ptsA*::*kan* strain grew twice as fast as the wild type, at close to the *in silico*-predicted optimal rate on DHA (Fig. 4A). The specific DHA uptake rate was likewise increased by 60% (Fig. 4B). Removing the kanamycin resistance cassette of the Δ*ptsA*::*kan* strain (i.e., Δ*ptsA*) decreased the growth rate to a value similar to the one measured for the wild-type strain on DHA (Fig. 4A). This means that PtsA is not itself involved in the overexpression of genes encoding the GLD and FSA pathways. Overall, these data are consistent with our previous results that overexpressing *fsaB* increases the DHA uptake rate. However, glycerol was only slightly accumulated in the Δ*ptsA*::*kan* strain (Fig. 4C), in contrast to what has been observed in the strain overexpressing *gldA*. This is consistent with the upregulation of *glpK* along with genes involved in *sn*-glycerol 3-phosphate (G3P) catabolism (i.e., *glpABC* and *glpQ*) and transport (*glpT*) (Table 3 and Data File S1) and, thus, with the use of the GLD pathway. The remaining question is why *glpK* is upregulated in the Δ*ptsA*::*kan* strain. This cannot be due only to the overexpression of *gldA*, since *glpK* was not upregulated in the wild-type strain that only overexpressed *gldA*.

**TABLE 3:** Functional classification of genes with statistically significant decreases and increases in mRNA level in E. coli BW25113 Δ*ptsA* and WT strains^*a*^

Category and expression status	GO concerned	*P* value	Genes involved
Overexpressed			
Secondary metabolism	GO:0019563; glycerol catabolic process	3.82E−11	*gldA*, *hybA*, *glpK*, *glpC*, *glpB*, *glpD*, *glpA*
	GO:0045333; cellular respiration	2.90E−19	*frdB*, *dmsA*, *dmsB*, *dmsC*, *hybB*, *fdnG*, *fdnH*, *frdA*, *frdC*, *frdD*, *hyaB*, *hybO*, *napB*, *napC*, *narG*, *narH*, *nirB*, *nirD*, *napA*, *glpD*, *glpA*, *glpB*, *glpC*, *ndh*, *hybA*, *hybC*, *hyaC*, *yjjI*, *cydA*, *cydB*
	GO:0071941; nitrogen cycle metabolic process	1.75E−09	*argF*, *napC*, *glnL*, *glnG*, *nirB*, *narG*, *nirD*, *nirC*, *narJ*, *narK*, *narH*, *napA*, *nrfA*
	GO:0019394; glucarate catabolic process	7.97E−06	*garL*, *gudD*, *garR*, *garK*
Response to stimuli	GO:0046688; response to copper ion	1.14E−08	*cusS*, *copA*, *cusA*, *cusB*, *cusC*, *cusF*, *cusR*, *yobA*, *cueO*
	GO:0009432; SOS response	1.74E−07	*recA*, *recN*, *sulA*, *umuC*, *umuD*, *yebG*, *recX*, *dinI*, *lexA*, *dinD*, *dinG*
Transport	GO:0015886; heme transport	2.41E−08	*ccmA*, *ccmD*, *ccmB*, *ccmC*, *ccmF*, *dppF*, *ccmE*, *dppC*
	GO:0015675; nickel cation transport	2.05E−05	*nikB*, *nikC*, *nikE*, *nikD*, *nikA*
	GO:0006857; oligopeptide transport	8.02E−04	*dppC*, *dppF*, *oppB*, *oppC*, *oppF*, *oppD*, *oppA*
Underexpressed			
Iron	GO:0055072; iron ion homeostasis	7.40E−15	*fepA*, *efeB*, *cirA*, *yqjH*, *fhuF*, *bfr*, *iscU*, *fhuA*, *fhuB*, *fhuC*, *fhuD*, *feoA*, *fepB*, *fes*, *fhuE*, *fecI*, *fecR*, *fepD*, *fepG*, *fepC*, *fiu*
	GO:0016226; iron-sulfur cluster assembly	2.05E−06	*iscS*, *iscU*, *erpA*, *sufB*, *sufD*, *sufA*, *sufC*, *sufE*, *sufS*
Secondary metabolism	GO:0009447; putrescine catabolic process	3.95E−10	*patA*, *patD*, *puuE*, *puuA*, *puuD*, *puuP*, *puuR*, *puuC*, *puuB*
	GO:0009065; glutamine family amino acid catabolic process	1.41E−05	*putA*, *astE*, *astD*, *astC*, *astB*, *astA*, *gabD*, *aldA*
	GO:0006790; sulfur compound metabolic process	7.66E−08	*prpB*, *prpD*, *prpE*, *prpR*, *fadH*, *fadI*, *fadE*, *fadM*, *fadD*, *fadA*, *fadB*, *yqeF*
Tricarboxylic acid	GO:0006099; tricarboxylic acid cycle	4.17E−09	*sdhC*, *sdhB*, *acnA*, *gltA*, *fumA*, *fumC*, *sdhA*, *aceB*, *aceA*, *aceK*, *sucA*, *prpC*, *prpD*
Fatty acids	GO:0009062; fatty acid catabolic process	4.62E−09	*prpB*, *prpD*, *prpE*, *prpR*, *fadH*, *fadI*, *fadE*, *fadM*, *fadD*, *fadA*, *fadB*, *yqeF*
	GO:0009712; catechol-containing compound metabolic process	8.28E−08	*entA*, *entC*, *entE*, *entH*, *entF*, *entB*, *fes*, *paaJ*
Hydrogen sulfide	GO:0070814; hydrogen sulfide biosynthetic process	1.25E−07	*astE*, *astD*, *astC*, *astB*, *astA*, *gabD*
Enterobactin	GO:0042930; enterobactin transport	3.10E−06	*fepA*, *fepD*, *fepG*, *fepC*, *fepB*, *entS*

a Levels in E. coli BW25113 Δ*ptsA* strain in M9-DHA medium were compared to those for E. coli BW25113 WT strain in M9-DHA medium. The Clusters of Orthologous Groups (COG) were used for grouping.

In the Δ*ptsA*::*kan* strain, genes coding for the DAK pathway were not upregulated, while genes encoding the glucarate pathway (used for glycolate assimilation) were highly expressed (7-fold on average) (Fig. 2 and Data File S1). Surprisingly, several genes associated with anaerobic or O_2_-limited conditions were upregulated (Table 3 and Data File S1). Several operons encoding enzymes in anaerobic respiratory chains (46, 50), i.e., (i) anaerobic dehydrogenases (GlpABC, FdnGHI, HyaABC, and HybABC), (ii) anaerobic terminal reductases (NarG, NapABCGH, NirBD, FrdABCD, and DmsABC), and (iii) microaerobic terminal oxidase (CydAB), were upregulated. All of these dehydrogenases are theoretically able to transfer electrons to each of the terminal reductases or oxidases, provided the enzymes react with the same type of quinone. This may provide the Δ*ptsA*::*kan* strain with a large variety of respiratory chains (46) to fulfill its energy requirements. Second, genes encoding enzymes in the tricarboxylic acid cycle and involved in fatty acid degradation were downregulated along with genes involved in putrescine catabolism. These genes are known to be repressed under anaerobic conditions (51–53). However, no change in the expression of Fnr, the major regulator governing the physiological switch between aerobic and anaerobic growth conditions and controlling the expression of all these genes (54, 55), was observed. Altogether, these data suggest that the Δ*ptsA*::*kan* strain adopts anaerobic growth behavior in a fully aerated medium, taking advantage of all available sources of carbon to grow on DHA while meeting its energetic needs.

In sum, these results demonstrate that optimal growth on DHA can be achieved by releasing hierarchical constraints on DHA metabolism, opening additional routes for its assimilation. However, the regulation mechanisms involved in the activation of anaerobic genes in the Δ*ptsA*::*kan* strain remain to be elucidated.

### Concluding remarks.

This study shows that aerobic DHA metabolism in E. coli is far from optimal because of chemical, hierarchical, and possibly allosteric constraints. However, our results show that removing hierarchical constraints optimizes growth on DHA. Beyond contributing to a better system-level understanding of DHA metabolism, these results are likely to accelerate the development of microbial biocatalysts. Enabling the rational design of strains that use DHA efficiently should improve biotechnological applications involving DHA as an intermediate, a well-known example being the bioconversion of glycerol into value-added chemicals (56). This should also facilitate research aiming at constructing synthetic methylotrophs (i.e., engineering of nonnative methylotrophs for methane and methanol-based production of chemicals) for which DHA can be an intermediate (47, 57).

## MATERIALS AND METHODS

### Bacterial strains and plasmids.

Escherichia coli K-12 BW25113 was selected as the model wild-type strain. Single-deletion mutants were taken from the Keio collection (49), and the Δ*dhaKLM* strain was constructed using the gene deletion method described previously (58). The *dhaKLM* operon was replaced by a Cm^r^ cassette using the primers listed in Table 2. All of the antibiotic resistance cassettes were removed by FLP recombination. The plasmids listed in Table 2 were purchased from BaseClear (Leiden, Netherlands). Cells were transformed according to the rubidium chloride protocol (59). All of the strains are listed in Table 2, and genetic modifications were checked by PCR.

### Growth conditions.

E. coli was cultivated overnight at 37°C with agitation at 220 rpm in LB broth (10 g tryptone, 5 g yeast extract, 10 g NaCl per liter) with appropriate antibiotics (100 μg/ml ampicillin or 50 μg/ml kanamycin) and 0.25 mM isopropyl β-d-1-thiogalactopyranoside (IPTG) if needed. The optical density at 600 nm (OD_600_) was measured by spectrophotometry, and 5-ml samples of preculture were centrifuged at 8,000 × *g* for 5 min. The pellets were resuspended in modified M9 medium (3.48 g/liter Na_2_HPO_4_·12H_2_O, 0.606 g/liter KH_2_PO_4_, 0.102 g/liter NaCl, 0.408 g/liter NH_4_Cl, 0.49 g/liter MgSO_4_, 4.38 mg/liter CaCl_2_, 15 mg/liter Na_2_EDTA·2H_2_O, 4.5 mg/liter ZnSO_4_·7H_2_O, 0.3 mg/liter CoCl_2_·6H_2_O, 1 mg/liter MnCl_2_·4H_2_O, 1 mg/liter H_3_BO_3_, 0.4 mg/liter Na_2_MoO_4_·2H_2_O, 3 mg/liter FeSO_4_·7H_2_O, 0.3 mg/liter CuSO_4_·5H_2_O, 0.1 g/liter thiamine) with no carbon source.

The cultures were incubated at 37°C and 220 rpm in 250-ml baffled shake flasks containing 50 ml of M9 medium supplemented with DHA (99.9% purity; Merck) at a final concentration of 15 mM. Cells were inoculated at an initial OD_600_ of 0.05, and growth was analyzed every 2 h for 48 h using a Thermo Genesys6 spectrophotometer (Thermo Scientific). Seven hundred fifty microliters of medium was collected and centrifuged at 8,000 × *g* for 3 min. Two hundred fifty microliters of supernatant was stored at −20°C before NMR or high-performance liquid chromatography (HPLC) analysis.

### Sampling, RNA extraction, and microarray procedures.

Cells were grown in duplicate under the same conditions as those described above. When an OD_600_ around 0.5 was reached (see Fig. S3 in the supplemental material), the cells were harvested and immediately frozen in liquid nitrogen. At such OD cells are in exponential growth phase, and DHA and formate are present in the medium for all of them (Fig. S3). For the WT strain, as the sampling time occurs during the night, the cultivation was diluted the next day in a fresh modified M9 medium at an OD of 0.2, and sampling was done at an OD_600_ of around 0.5. Total RNA was extracted by following the Qiagen RNeasy minikit procedure and quantified using a NanoDrop spectrophotometer. Double-stranded complementary DNA (cDNA) synthesis and array processing were performed using the Agilent Technologies one-color microarray-based gene expression analysis protocol. The images were analyzed with the software DEVA (v1.2.1). All array procedures were performed using the GeT-Biopuces platform (http://get.genotoul.fr/). For the wild-type strain, gene expression of batch cultures with DHA was expressed relative to the ratio of gene expression of chemostatic growth with glucose at low (μ = 0.1 h^−1^) and high (μ = 0.6 h^−1^) growth rates (data from reference [60]). This allowed us to focus only on genes whose level of expression changes in relation to the nature of the substrate while avoiding highlighting of genes whose expression changes because of the very different growth rate. For the Δ*dhaKLM* and Δ*ptsA*::*kan* mutants, gene expression on DHA was compared to the wild type’s gene expression on DHA. Gene ontology analyses were performed using EcoCyc (61).

### NMR and HPLC analysis.

NMR analyses were performed on an Avance III 800-MHz spectrometer (Bruker, Rheinstetten, Germany) equipped with a 5-mm QPCI cryogenic probe head at 280K. Supernatants were analyzed by quantitative ^1^H one-dimensional NMR at 280K using a zgpr30 sequence with water presaturation. A total of 32 scans were accumulated after 8 dummy scans. The time domain function (the FID) was converted to the frequency domain function (the spectrum) by Fourier transform. The phase of the spectra was adjusted manually, the baseline was corrected automatically, and the spectra were aligned using the signal from 3-trimethylsilylpropionic-2,2,3,3-d4 acid sodium salt (TSP-d4), an internal standard, with the Bruker software TopSpin (v3.5). Propane-1,2,2,3-tetrol was identified using 2D heteronuclear single quantum correlation spectroscopy (HSQC) and heteronuclear multiple-bond correlation spectroscopy (HMBC) spectra. The HSQC experiment was acquired with 2,000 by 1,024 points for a spectral width of 13.95 by 140 ppm in the ^1^H and ^13^C dimensions, respectively. It was processed with 4,000 by 1,024 points. The HMBC experiment was acquired with 2,000 by 1,024 points, for a spectral width of 13.95 by 220 ppm in the ^1^H and ^13^C dimensions, respectively. It was processed with 8,000 by 1,024 points. The HSQC and HMBC data were acquired with 16 scans per increment. Both 2D spectra were calibrated in the frequency domain by setting the peak from TPS-d4 to 0 ppm in both dimensions. The spectra were processed using TopSpin 3.5.

For transcriptomic analysis, HPLC analyses were performed using a column made of an H^+^ chromatography resin (Zorbax-C_18_; Agilent Technologies). A solution of sulfuric acid (5%, vol/vol) was used as the eluent at a flow rate of 0.5 ml min^−1^ and a volume of injection of 20 μl. The oven temperature was set to 48°C. Ranges of standards of glycerol, formate, and DHA were realized in order to quantify extracellular metabolites. Retention times in minutes for each compound were the following: glycerol, 16.6 (refractometry detection); DHA, 16.3 (UV detection); and formate, 16.9 (UV detection). Calibration curves of these three compounds were established and used to calculate their concentrations in the culture supernatants.

### Calculation of growth, substrate uptake, and degradation rates.

We developed a mathematical model to infer quantitative growth and exchange flux information from the measured time-dependent concentrations of biomass and extracellular metabolites. The general model, which accounts for the nonenzymatic degradation of substrates or products, relates changes of concentrations to fluxes using the following system of ordinary differential equations:

(1) $$\frac{\mathrm{dX}}{\mathrm{dt}}=\mu \cdot X$$

(2) $$\frac{dM_{i}}{\mathrm{dt}}=-k\cdot M_{i}+X\cdot q_{M_{i}}$$

where *X* is the biomass concentration (g_DW_ liter^−1^), *μ* is the growth rate (h^−1^), and *M_i_* is the concentration of exchanged metabolite *i* (mmol liter^−1^) with a degradation constant, *k* (h^−1^), and exchange flux, *q_M_i__* (mmol g_DW_^−1^ h^−1^). Integrating equations 1 and 2 provides the following analytical functions:

(3) $$X\left(t\right)=X_{0}\cdot e^{\mu t}$$

(4) $$M_{i}\left(t\right)=q_{M_{i}}\cdot \frac{X_{0}}{\mu +k}\cdot \left(e^{\mu t}-e^{-\mathrm{kt}}\right)+M_{i}^{0}\cdot e^{-\mathrm{kt}}$$

This formalism has been implemented in PhysioFit, a flexible R program that allows growth rates and exchange fluxes to be quantified by fitting time variations of extracellular metabolite and biomass concentrations using the *nlsic* algorithm (62). PhysioFit includes options to account (or not) for the degradation of extracellular compounds and a lag phase before cells start to grow, and it implements sensitivity analyses to evaluate the precision of the estimated fluxes. PhysioFit is provided open source at https://github.com/MetaSys-LISBP/PhysioFit.

A conversion factor was used to obtain cellular dry weights (DWs) from OD_600_ (0.37 g_DW_/OD_600_) and calculate specific DHA uptake rates (mmol g_DW_^−1^ h^−1^), formate production yields (mmol g_DW_^−1^ h^−1^), and biomass yields (g mol^−1^). Experiments were performed in triplicate to calculate averages and standard deviations.

### *In silico* analysis of DHA metabolism.

*In silico* analyses of DHA metabolism were carried out with the E. coli genome-scale metabolic model iJO1366 (37) (Biomodels identifier MODEL1108160000) constrained with experimental uptake fluxes of the wild-type strain (DHA = 5.2 mmol g_DW_^−1^ h^−1^, formate = 3.2 mmol g_DW_^−1^ h^−1^, glycolate = 1.0 mmol g_DW_^−1^ h^−1^, and acetate = 0.1 mmol g_DW_^−1^ h^−1^), using Sybil package (v2.1.2) (63) of the R environment (v3.2.4) (64, 65). The model and all the scripts used to run calculations are available at https://github.com/MetaSys-LISBP/Peiro_2019 under the GPLv3 open source license to ensure reproducibility and reusability.

### Data availability.

Gene expression data have been deposited in the ArrayExpress database at EMBL-EBI (www.ebi.ac.uk/arrayexpress) under accession number E-MTAB-7666.

## Supplementary Material

Supplemental file 1

Supplemental file 2
