# Supplementary material for: Chemical and Metabolic Controls on Dihydroxyacetone Metabolism Lead to Suboptimal Growth of Escherichia coli
Source: Appl Environ Microbiol. 2019 Jul 18;85(15):e00768-19. doi: 10.1128/AEM.00768-19 (PMC6643234; doi:10.1128/AEM.00768-19)
Supplement: Supplemental file 1 [file AEM.00768-19-s0001.pdf]

SUPPLEMENTAL MATERIALS

Chemical and hierarchical controls on dihydroxyacetone metabolism lead to a suboptimal growth of *E. coli*.

Camille Peiro[1], Pierre Millard[1], Alessandro de Simone[1], Edern Cahoreau[1], Lindsay Peyriga[1], Brice Enjalbert[1], Stéphanie Heux[1#]

1 LISBP, Université de Toulouse, CNRS, INRA, INSA, Toulouse, France

# Address correspondence to Stéphanie Heux, heux@insa-toulouse.fr

(A)

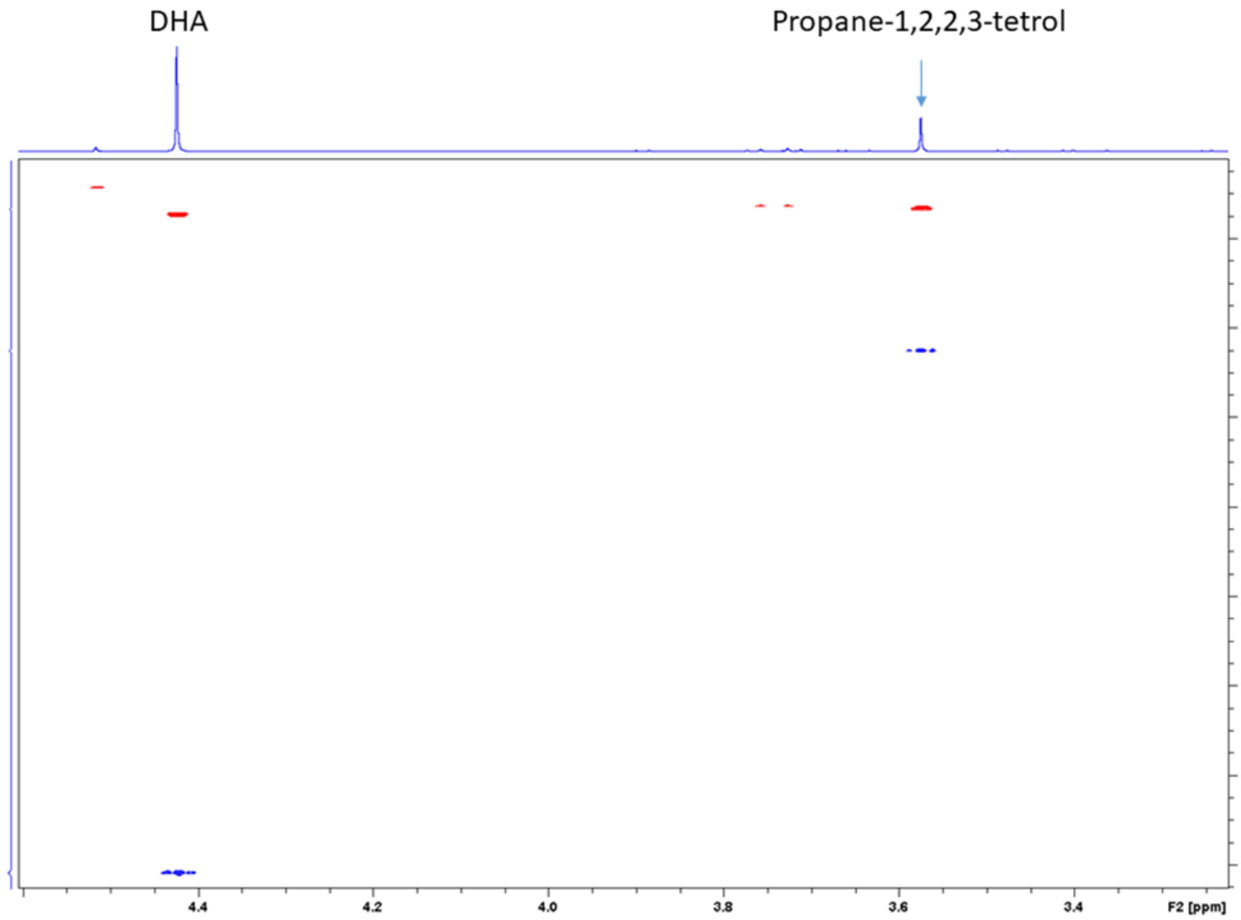

(B)

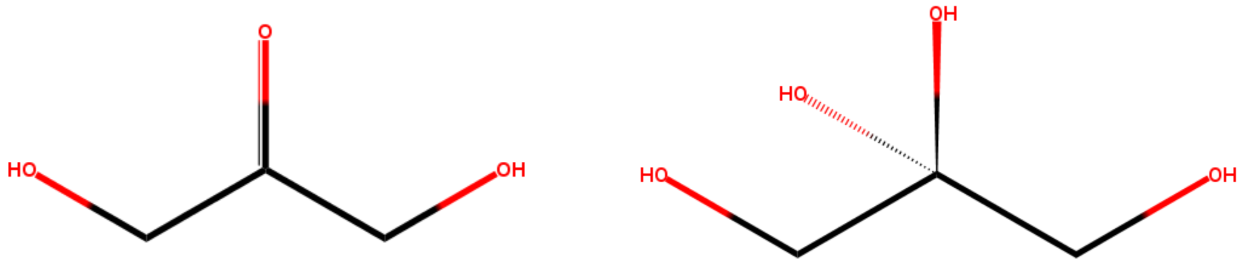

Figure S1: Identification of propane-1,2,2,3-tetrol by 2D-NMR analyses in a sample of DHA in M9 medium with 5X diluted salts. (A)Overlay of Heteronuclear Single Quantum Correlation spectroscopy (HSQC) experiment in red and Heteronuclear Multiple Bonds Correlation spectroscopy (HMBC) experiment in blue. (B)DHA (left) and propane-1,2,2,3-tetrol (right) condensed formula.

The HSQC experiments enables to see one bond correlation between the ^{1}H and ^{13}C atoms called $^{1}J_{CH}$ and the HMBC experiments exhibits $^{2}J_{CH}$ to $^{3}J_{CH}$, i.e. long distance correlations between a ^{1}H and a ^{13}C. From HSQC acquisitions (Figure S1A in red), we observed that protons at 4.4 ppm correlates with carbon resonance at 54.4 ppm and ^{1}H 3.6ppm signal correlates with 53.0 ppm carbon signal. Knowing that the DHA molecular structure (Figure S1B), we can assume that the peak at 54.4 ppm corresponds to a hydroxyl group. Likewise, we can assume that the peak at 53.0 ppm corresponds also to a hydroxyl group with a very slight deshielded. From the HMBC spectrum (Figure S1A in blue), a correlation peak between ^{1}H at 4.4 ppm and ^{13}C at 200 ppm was observed corresponding to the ketone group. For the unknown molecule, a correlation peak between ^{1}H at 3.6 ppm and carbon at 82.9 ppm was observed (Figure S1A). This carbon chemical shift can be due to a deshielded carbon bound to two hydroxyl

groups. According to these data, we suggest that the unknown molecule is most probably a derived form of DHA. This is in accordance with the results of Yaylayan and colleagues (1) who described that in water DHA can be hydrated into a secondary form i.e. propane-1,2,2,3-tetrol (or dihydroxyacetone monomer hydrate) (Figure S1B).

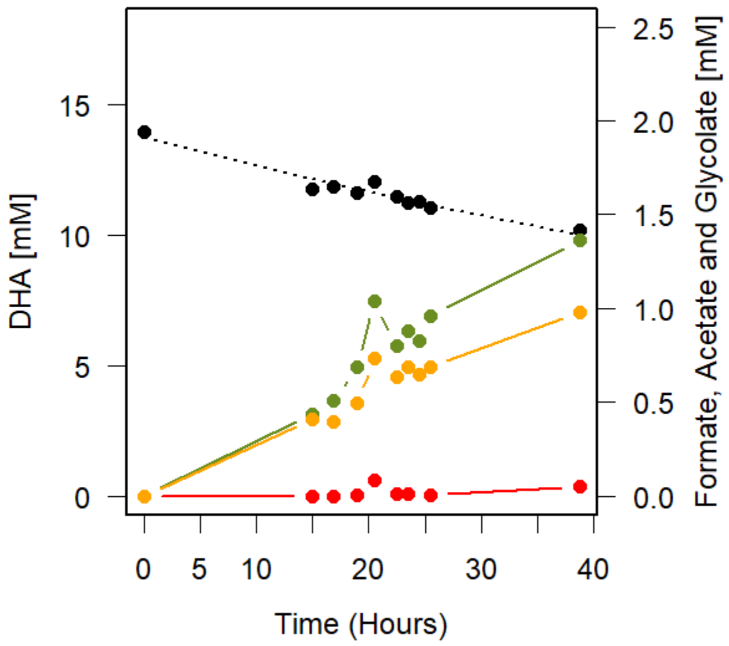

Figure S2: Time course analysis of the fate of DHA in non-inoculated modified M9 medium at 37°C, 250 rpm shaking. Kinetic of degradation of the DHA (experimental (black points) and fitted (dashed black lines) into formate (green), glycolate (orange) and acetate (red). One out of three experiments is shown.

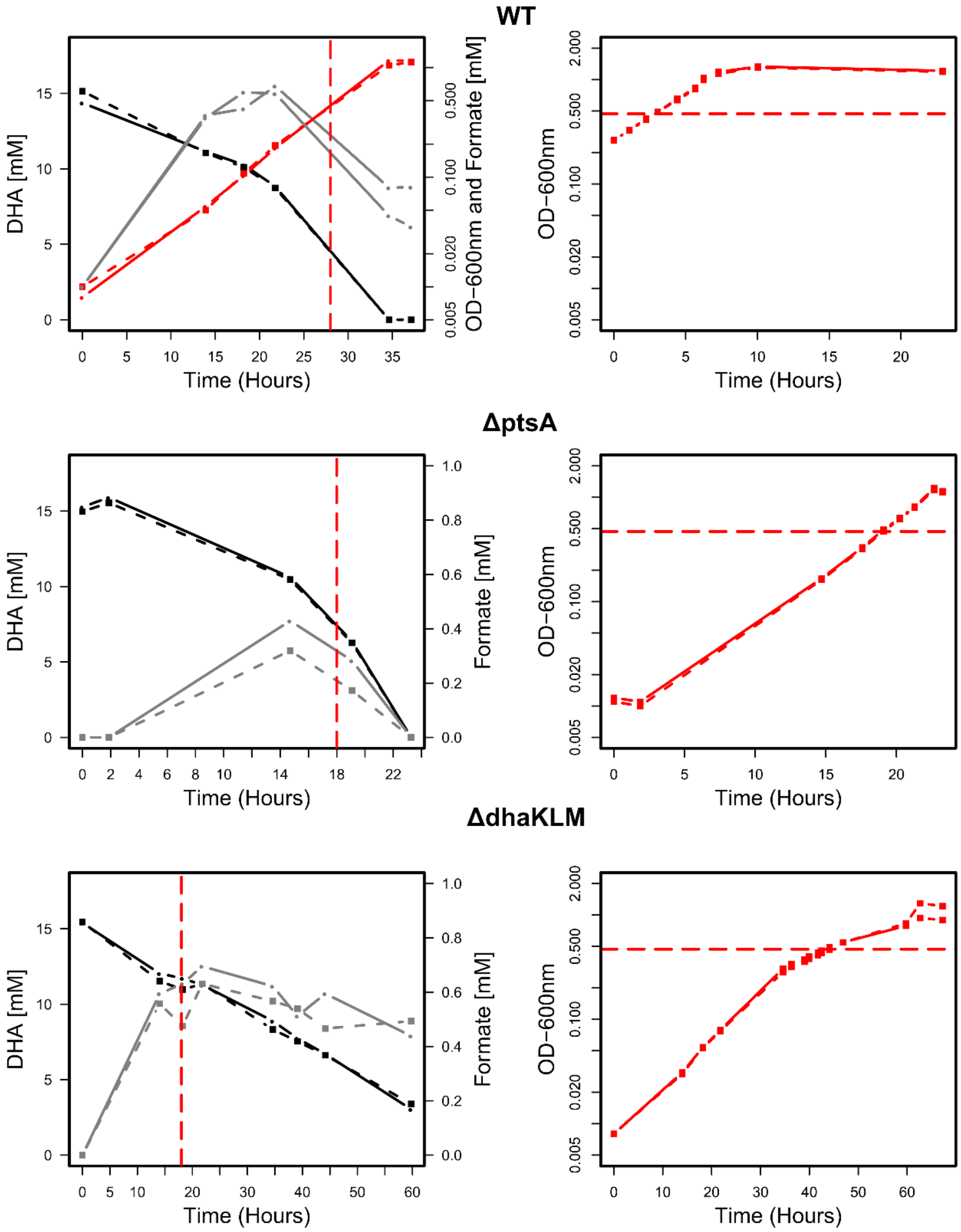

Figure S3: Time course analysis of the fate of DHA, in *E. coli* BW25113 strain (WT) and in the deleted for *ptsA* (Δ*ptsA*) and *dhaKLM* (Δ*dhaKLM*) strains. Cells were grown modified M9 medium with 15 mM of DHA at 37°C, 250 rpm shaking. DHA (black), formate (grey) and growth (red). Vertical dashed red lines indicate the sampling time point for transcriptomic analysis. The axis of the growth plot are in logarithmic scale. For the WT strain, the sampling for transcriptomic analysis was done the next day from the same cultivation inoculated in a fresh medium at OD_{600nm} = 0.2. (n=2 biological replicates). Supernatant were analysed by HPLC and NMR. Results obtain by HPLC are presented. Growth rate and specific DHA uptake rate for each strains are provided in figure 4.

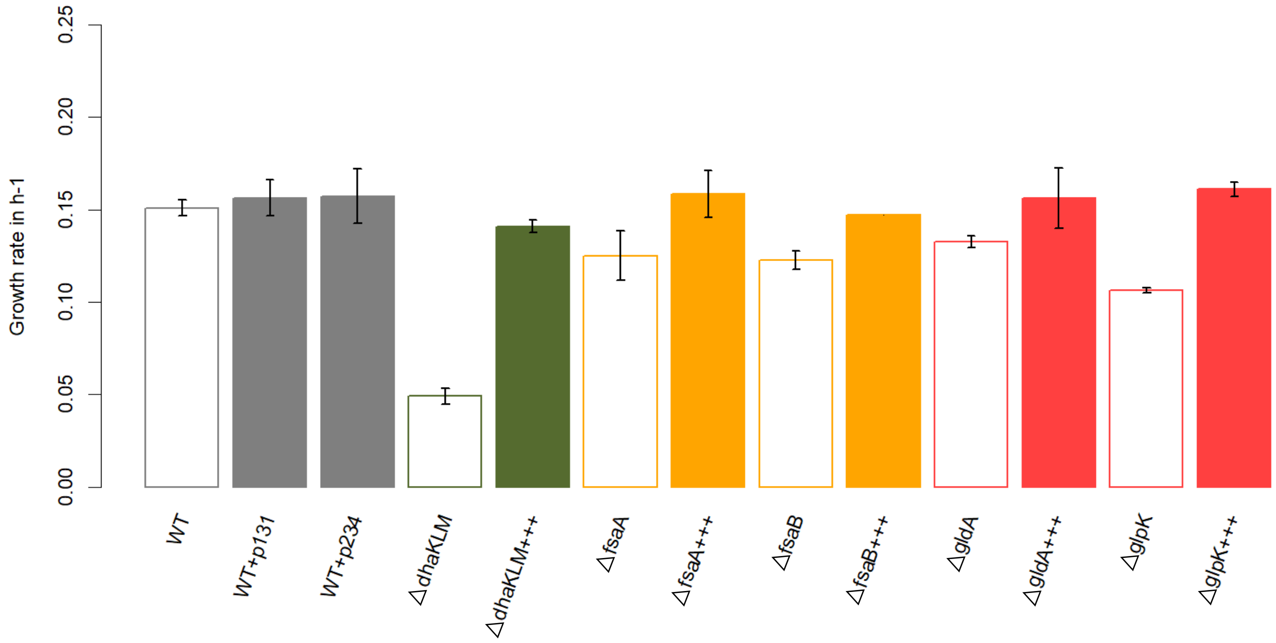

Figure S4: Growth rate of the deleted (Δ*gene*) and complemented (Δ*gene*+++) strain. n = 2 biological replicates for the complemented stain and 3 biological replicates for the deleted strains. Cells were grown modified M9 medium with 15 mM of DHA at 37°C, 250 rpm shaking. Please refer to Table 2 for details on each strain.

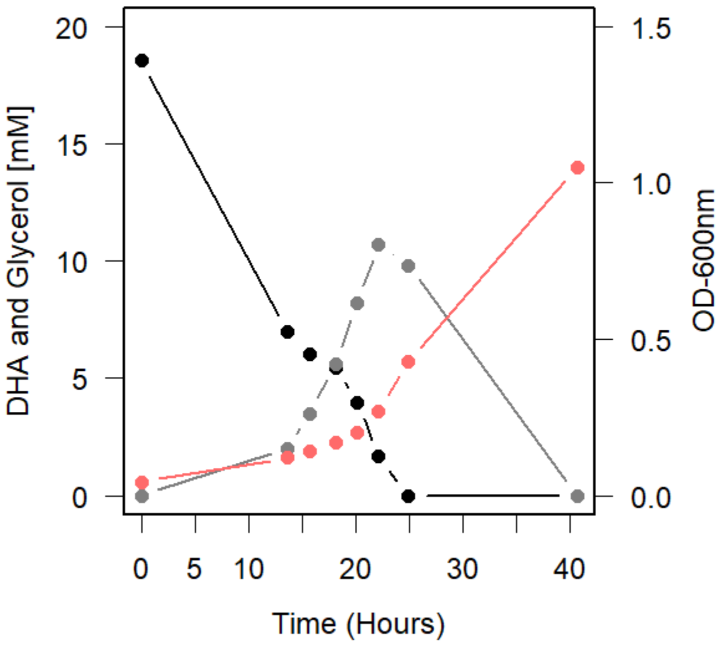

Figure S5: Time course analysis of the fate of DHA in an *E. coli* BW25113 strain overexpressing *gldA*. Cells were grown modified M9 medium with 15 mM of DHA at 37°C, 250 rpm shaking. DHA (black), glycerol (gray) and growth (red). One over three experiment is shown.

REFERENCES

1. Yaylayan VA, Harty-Majors S, Ismail AA. 1999. Investigation of DL-glyceraldehyde–dihydroxyacetone interconversion by FTIR spectroscopy. Carbohydrate Research 318:20–25.
